# Preoperative Naples prognostic score is a reliable prognostic indicator for newly diagnosed glioblastoma patients

**DOI:** 10.3389/fonc.2022.775430

**Published:** 2022-08-16

**Authors:** Junhong Li, Wanchun Yang, Yunbo Yuan, Mingrong Zuo, Tengfei Li, Zhihao Wang, Yanhui Liu

**Affiliations:** Department of Neurosurgery, West China Hospital of Sichuan University, Chengdu, China

**Keywords:** naples prognostic score, glioblastoma, inflammation, nutrition, prognosis

## Abstract

**Background:**

Glioblastoma (GBM) accounts for approximately 80% of malignant gliomas and is characterized by considerable cellularity and mitotic activity, vascular proliferation, and necrosis. Naples prognostic score (NPS), based on inflammatory markers and nutritional status, has a prognostic ability in various cancers. In the current study, we aim to explore the prognostic value of operative NPS in GBM patients and compare the prognostic ability between NPS and controlling nutritional status (CONUT).

**Materials and methods:**

The retrospective analysis was carried out on consecutive newly diagnosed GBM patients who had underwent tumor resection at West China Hospital from February 2016 to March 2019. All statistical analyses were conducted using SPSS software and R software.

**Results:**

A total of 276 newly diagnosed GBM patients were enrolled in the current study. Overall survival (OS) (*p* < 0.001) and tumor location (*p* = 0.007) were significantly related to NPS. Serum albumin concentrate, cholesterol concentrate, neutrophil-to-lymphocyte ratio, lymphocyte ratio, and CONUT score were all significantly associated with NPS (*p* < 0.001). The Kaplan–Meier curve indicated that NPS (log-rank test, *p* < 0.001) and CONUT score (log-rank test, *p* = 0.023) were significantly associated with OS. Multivariate Cox regression revealed that both NPS and CONUT score served as independent prognostic indicators. The prognostic model with NPS had the strongest prognostic capability and best model-fitting.

**Conclusion:**

In the current study, NPS is found as an independent prognostic indicator for patients with newly diagnosed GBM, and the prognostic ability of NPS is superior to CONUT score.

## Introduction

Gliomas are the most common primary malignant brain tumors in adults, which can occur anywhere in the central nervous system but primarily occur in the brain and originate in the glial tissue (1). Glioblastoma (GBM), the World Health Organization (WHO) grade 4 glioma, accounts for approximately 80% of malignant gliomas and is characterized by considerable cellularity and mitotic activity, vascular proliferation, and necrosis (2). The median overall survival (OS) is about 15 months in patients with this incurable disease, even after receiving standardized radiotherapy and temozolomide (TMZ), and there also remains a very high risk of recurrence (3). Despite considerable effort, little progress has been made toward prolonged survival in GBM (4).

In 1863, Rudolf Virchow firstly introduced the concept “inflammation and cancer” based on the observation on leukocytes in neoplastic tissues (5). In recent years, researchers have found that inflammation plays a crucial role in tumorigenesis, which is also considered as a hallmark feature of cancer development and progression (6–8). Systemic inflammatory response, mainly produced by peripheral immune cells, could indirectly reflect the severity of local malignancy. Peripheral blood neutrophil, lymphocyte, monocyte, and platelet are widely used as systemic inflammatory markers for predicting prognosis and evaluating therapeutic response of tumors (9–11). Nutrition is also proposed to play an essential role in cancer progression based on the principle that it can also cause oxidative stress, augment a cascade of molecular reactions in cells, and alter the metabolic state of tissues (12, 13). Patients with wasting diseases like malignancies usually have weak physical conditions. Nutritional markers like albumin, hemoglobin, and BMI have been proven to be effective predictive indicators for disease progression and play a prognostic role (14, 15).

Naples prognostic score (NPS), based on inflammatory markers and nutritional status, was firstly introduced in the research of colorectal cancer by Galizia et al. (16) So far, the prognostic significance has been validated in various cancers (17–20). Controlling nutritional status (CONUT) score is also a useful nutritional marker to evaluate nutritional status and predict prognosis in cancer patients, which is similar in composition with NPS (21–23). Recent studies have reported that both preoperative and postoperative CONUT score serve as an independent prognostic indicator for GBM patients (24, 25). In the current study, we aim to explore the prognostic value of preoperative NPS in GBM patients and compare the prognostic ability between NPS and CONUT.

## Materials and methods

### Patients

The retrospective analysis was carried out on consecutive newly diagnosed GBM patients who had underwent tumor resection at West China Hospital from February 2016 to March 2019. All the patients underwent a craniotomy on GBM with gross total resection (GTR) or subtotal resection (STR), and their baseline clinical data were retrieved from the hospital information system. The extent of resection was determined by surgical records and postoperative magnetic resonance imaging (MRI) and computed tomography (CT). The pathological diagnosis criteria followed the 2016 WHO classification of CNS tumors. These patients were followed up until March 2021.

The exclusion criteria were as follows: (1) younger than 18 years; (2) partial resection or biopsy; (3) absence of definite pathological diagnosis; (4) incomplete baseline clinical data; (5) receiving adjuvant therapy including radiotherapy, chemotherapy, and corticosteroid before surgery; (6) absence of preoperative MRI; (7) presence of history of infectious diseases, blood system diseases, or other malignancies before surgery; (8) recurrent GBM; and (9) lost to follow-up.

### Parameter assessment

The following clinical variables were retrieved from the hospital information system: (1) demographics: age at diagnosis and gender; (2) preoperative conditions: Karnofsky performance status (KPS) score and presence of preoperative seizures; (3) imaging characteristics: tumor locations and maximum diameter. The maximum diameter was defined as the longest distance of the maximum section in gadolinium-enhanced T1 sequence. If there was no significant tumor enhancement, the T2 FLAIR sequence was applied; (4) pathological markers: Ki-67 index and the status of isocitrate dehydrogenase-1 (IDH-1); (5) conditions of adjuvant therapy; (6) blood test results: serum albumin, cholesterol, neutrophil count, lymphocyte count, and monocyte count. Routine blood test was conducted within 3 days before operation in our center.

**Figure 1:**
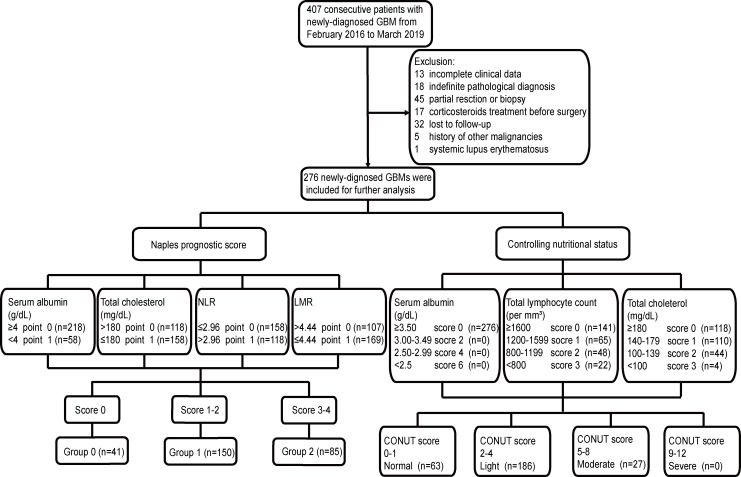
Flowchart of the current study. GBM, glioblastoma; NPS, Naples prognostic score; CONUT, Controlling Nutritional Status; NLR, neutrophil-to-lymphocyte ratio; LMR, lymphocyte-to-monocyte ratio.

NLR was defined as neutrophil count/lymphocyte count, and LMR was defined as lymphocyte count/monocyte count. NPS was calculated from serum albumin and cholesterol. NLR and LMR were built upon the research of Galizia et al. (16) CONUT score was calculated based on the count of serum albumin, cholesterol, and lymphocyte according to the previous study (21) (**Figure 1**).

After initial treatment, the patients were followed up every 3–6 months. OS was defined as the duration from the date of operation to death or the end of the observation period.

### Statistical analysis

All statistical analyses were conducted using SPSS software (Version 22.0, IBM Co., Armonk, NY, USA) and R software (Version 3.6.1). Continuous variables were presented as mean ± standard deviation (SD) or median with interquartile range (IQR), and categorical variables were presented as frequency and percentage. Categorical variables were compared using the chi-square test. Data that conformed to the normal distribution were compared using Student’s *t*-test; otherwise, Mann–Whitney *U* test or Kruskal–Wallis test was applied. Kaplan–Meier (K-M) curves were applied to calculate cumulative OS using the log-rank test. The Cox regression analyses were employed to determine the influences of risk factors for OS in GBM patients. Variables with *p*-value <0.1 in univariate analysis were included into backward stepwise multivariate Cox regression. Harrell’s concordance index (C-index) and Akaike information criterion (AIC) were calculated to evaluate prognostic models. Higher C-index indicated better predictive accuracy, while lower AICs indicated superior model-fitting (26, 27). A two-sided *p*-value <0.05 was referred to as statistically significant difference.

### Ethics statement

This study was approved by the Ethical Committee of Sichuan University and conducted according to the principles expressed in the Declaration of Helsinki. All patients and their authorized trustees were informed before surgery and signed their informed consent to use their clinical data for research purposes.

## Results

### Baseline characteristics

After screening (**Figure 1**), a total of 276 newly diagnosed GBM patients were enrolled in the current study, including 185 (67.0%) men and 91 (33.0%) women (**Table 1**). The average age at diagnosis was 53.41 years. Forty-four (15.9%) patients had seizures before surgery, and 102 (37.0%) patients had a better KPS score (>80). Detailed tumor locations and maximum diameter are listed in **Table 1**. As for postoperative treatment, 217 patients received the Stupp’s regimen that contained 42-day concomitant radiochemotherapy and subsequent 6–12 consecutive cycles of TMZ alone, whereas the other 59 patients did not receive adjuvant therapy or discontinued the treatment at an early stage due to various reasons. High Ki-67 index was detected in 121 (43.8%) patients, and 35 (12.7%) patients had IDH-1 mutation. CONUT score was divided into four groups according to the score system. There were 63 (22.8%), 186 (67.4%), and 27 (9.8%) patients, respectively, in the Normal group, Light group, and Moderate group. No patient was included in the Severe group. Based on the definition of NPS, 41 (14.9%), 150 (54.3%), and 85 (30.8%) patients were classified into Group 0, Group 1, and Group 2, respectively.

**Table 1:** Baseline clinical characteristics of glioblastoma patients in the current cohort.

Clinical Characteristic	Total (*n* = 276)	Naples Prognostic Score			*p*-value
		Group 0 (*n* = 41)	Group 1 (*n* = 150)	Group 2 (*n* = 85)
**Overall survival**	329 (193–550)	516 (282–885)	375 (198–547)	240 (128–428)	***<0.001***
**Age at diagnosis**	53.41 ± 14.24	50.98 ± 11.27	53.13 ± 14.89	55.07 ± 14.30	0.250
**Gender**					
Male	185 (67.0)	23 (56.1)	99 (66.0)	63 (74.1)	0.121
Female	91 (33.0)	18 (43.9)	51 (34.0)	22 (25.9)	
**Preoperative seizures**					
Yes	44 (15.9)	10 (24.4)	21 (14.0)	13 (15.3)	0.268
No	232 (84.1)	31 (75.6)	129 (86.0)	72 (84.7)	
**KPS**					
≤80	174 (63.0)	25 (61.0)	92 (61.3)	57 (67.1)	0.653
>80	102 (37.0)	16 (39.0)	58 (38.7)	28 (32.9)	
**Hemisphere**					
Right	130 (47.1)	21 (51.2)	65 (43.3)	44 (51.8)	0.456
Left	132 (47.8)	18 (43.9)	79 (52.7)	35 (41.2)	
Midline or bilateral	14 (5.1)	2 (4.9)	6 (4.0)	6 (7.1)	
**Location**					
Frontal lobe	98 (35.5)	18 (43.9)	56 (37.3)	24 (28.2)	***0.007***
Temporal lobe	54 (19.6)	6 (14.6)	18 (12.0)	30 (35.3)	
Parietal lobe	23 (8.3)	4 (9.8)	16 (10.7)	3 (3.5)	
Occipital lobe	7 (2.5)	2 (4.9)	3 (2.0)	2 (2.4)	
Insular lobe	10 (3.6)	1 (2.4)	7 (4.7)	2 (2.4)	
Other locations	84 (30.4)	10 (24.4)	50 (33.3)	24 (28.2)	
**Maximum diameter (mm)**					
<50	134 (48.6)	20 (48.8)	79 (52.7)	35 (41.2)	0.240
≥50	142 (51.4)	21 (51.2)	71 (47.3)	50 (58.8)	
**Adjuvant therapy**					
Yes	217 (78.6)	35 (85.4)	122 (81.3)	60 (70.6)	0.081
No or Undone	59 (21.4)	6 (14.6)	28 (18.7)	25 (29.4)	
**Ki-67**					
<30%	155 (56.2)	27 (65.9)	84 (56.0)	44 (51.8)	0.327
≥30%	121 (43.8)	14 (34.1)	66 (44.0)	41 (48.2)	
**IDH-1**					
Mutant	35 (12.7)	8 (19.5)	20 (13.3)	7 (8.2)	0.192
Wild type	241 (87.3)	33 (80.5)	130 (86.7)	78 (91.8)	
**Albumin (g/ml)**	4.28 (4.03–4.52)	4.52 (4.27–4.67)	4.32 (4.15–4.50)	3.96 (3.75–4.33)	***<0.001***
**Cholesterol (mg/dl)**	172.47 (149.36–195.96)	199.92 (186.00–216.36)	173.24 (150.23–198.38)	156.23 (132.25–168.99)	***<0.001***
**NLR**	2.56 (1.84–4.21)	1.62 (1.33–2.01)	2.35 (1.84–3.36)	4.03 (3.27–6.29)	***<0.001***
**LMR**	4.03 (2.91–5.30)	5.52 (4.89–6.94)	4.23 (3.13–5.53)	2.88 (2.07–3.66)	***<0.001***
**CONUT score**					
Normal	63 (22.8)	33 (80.5)	26 (17.3)	4 (4.7)	***<0.001***
Light	186 (67.4)	8 (19.5)	118 (78.7)	60 (70.6)	
Moderate	27 (9.8)	0 (0.0)	6 (4.0)	21 (24.7)	
Severe	0 (0.0)	0 (0.0)	0 (0.0)	0 (0.0)	

Data are presented as n (%), mean ± SD, or median (25th, 75th quartile).

Significant findings (p < 0.05) are expressed in bold and italic.

KPS, Karnofsky performance status; IDH-1, Isocitrate dehydrogenase-1; NLR, neutrophil-to-lymphocyte ratio; LMR, lymphocyte-to-monocyte ratio; CONUT, Controlling Nutritional Status.

### Associations between NPS and clinical variables

**Table 1** depicts the relationships between NPS and other clinical variables. OS (*p* < 0.001) and tumor location (*p* = 0.007) were significantly related to NPS, while other variables including age at diagnosis (*p* = 0.250), gender (*p* = 0.121), preoperative seizures (*p* = 0.268), KPS score (*p* = 0.653), hemisphere (*p* = 0.456), maximum diameter (*p* = 0.240), adjuvant therapy (*p* = 0.081), Ki-67 index (*p* = 0.327), and IDH-1 mutation status (*p* = 0.192) were not evidently connected with NPS. As for components of NPS, four peripheral markers were all significantly associated with NPS (*p* < 0.001). There were significant differences between NPS groups 0, 1, and 2 in CONUT score (*p* < 0.001).

### Prognostic value of NPS

**Figure 2:**
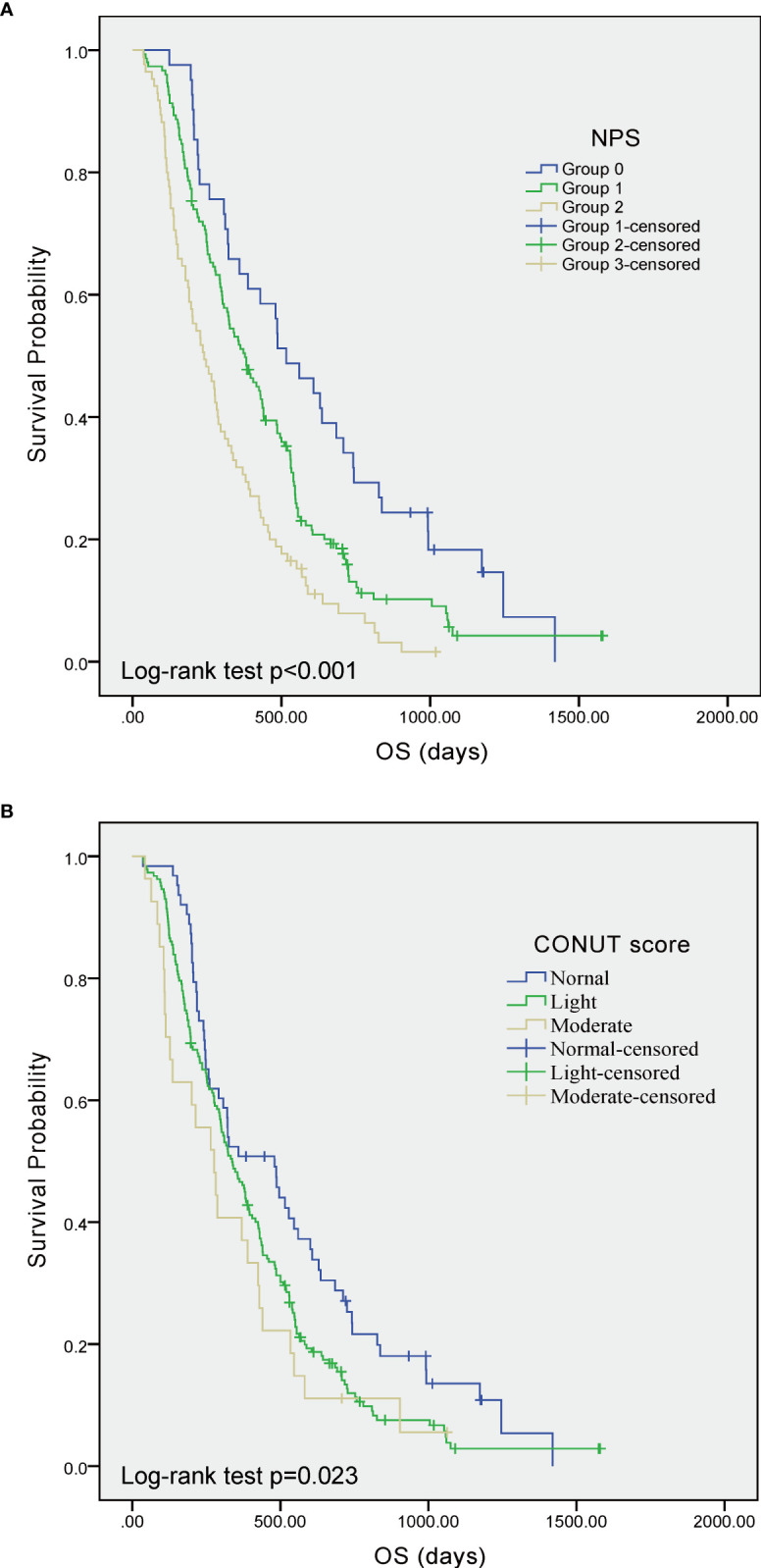
Kaplan–Meier curves showing overall survival of GBM patients stratified by value of NPS **(A)** and CONUT score **(B)**, respectively. GBM, glioblastoma; NPS, Naples prognostic score; CONUT, Controlling Nutritional Status; OS, overall survival.

As shown in **Figure 2**, the K-M curve was firstly employed to evaluate the prognostic significance of NPS and CONUT score in GBM patients. The results indicated that NPS (log-rank test, *p* < 0.001) and CONUT score (log-rank test, *p* = 0.023) were significantly associated with OS.

Cox regression analysis was used to further determine the prognostic roles of clinical variables (**Table 2**). In univariate Cox regression, elder patients, male, lower KPS score, longer maximum diameter, uncompleted adjuvant therapy, and IDH-1 wild type were significantly associated with poor prognosis. Tumor location was also evidently related to OS. Albumin ≥4 g/dl and cholesterol >180 mg/dl were in connection with favorable prognosis, while NLR >2.96 indicated poor prognosis. CONUT score and NPS were also significantly related to prognosis, and hazard ratios were raised accompanied by the increase of the score. Multivariate Cox regression indicated that both CONUT score and NPS served as independent prognostic indicators. Other independent prognostic indicators included age, adjuvant therapy, and IDH-1 status.

**Table 2:** Univariate and multivariate Cox regression for risk factors predictive of GBMs.

Clinical Variables		Univariate Analysis			Multivariate Analysis		
	HR	95% CI	*p*-value	HR	95% CI	*p*-value
**Age at diagnosis**	<55	Reference			Reference		
	≥55	1.929	1.491–2.496	***<0.001***	1.548	1.175–2.039	***0.002***
**Gender**	Male	Reference			Reference		
	Female	0.711	0.544–0.930	***0.013***	1.306	0.992–1.718	0.057
**KPS**	≤80	Reference			Reference		
	>80	0.743	0.573–0.963	***0.025***	0.795	0.606–1.042	0.097
**Hemisphere**	Right	Reference		0.104			
	Left	0.902	0.699–1.165	0.430			
	Midline or bilateral	1.674	0.942–2.975	0.079			
**Location**	Frontal lobe	Reference		***0.009***			0.339
	Temporal lobe	1.516	1.072–2.144	***0.019***	1.107	0.760–1.612	0.597
	Parietal lobe	0.966	0.599–1.558	0.887	1.319	0.808–2.152	0.268
	Occipital lobe	1.000	0.436–2.294	0.999	1.691	0.702–4.074	0.242
	Insular lobe	1.094	0.529–2.260	0.809	1.095	0.518–2.317	0.812
	Other regions	1.723	1.264–2.349	***0.001***	1.418	1.022–1.967	***0.037***
**Maximum diameter**	<50	Reference					
	≥50	1.338	1.042–1.717	***0.022***	1.135	0.864–1.490	0.362
**Preoperative seizures**	No	Reference					
	Yes	0.953	0.681–1.334	0.778			
**Adjuvant therapy**	Yes	Reference			Reference		
	No or Undone	1.790	1.326–2.416	***<0.001***	1.727	1.273–2.342	***<0.001***
**Ki67**	<30%	Reference					
	≥30%	1.109	0.864–1.424	0.416			
**IDH-1**	Mutant	Reference			Reference		
	Wild type	2.153	1.421–3.262	***<0.001***	1.792	1.162–2.762	***0.008***
**Albumin (g/dl)**	<4	Reference			Reference		
	≥4	0.625	0.484–0.805	***<0.001***	0.731	0.554–0.965	***0.027***
**Cholesterol (mg/dl)**	≤180	Reference			Reference		
	>180	0.561	0.431–0.731	***<0.001***	0.670	0.503–0.891	***0.006***
**NLR**	≤2.96	Reference			Reference		
	>2.96	1.610	1.193–2.172	***0.002***	1.168	0.854–1.598	0.331
**LMR**	≤4.44	Reference					
	>4.44	0.840	0.653–1.080	0.174			
**CONUT score**	Normal	Reference		***0.022***	Reference		***0.014***
	Light	1.429	1.051–1.943	***0.023***	1.300	0.953–1.774	0.098
	Moderate	1.795	1.116–2.888	***0.013***	2.086	1.287–3.380	***0.003***
**NPS**	Group 0	Reference		***<0.001***	Reference		***<0.001***
	Group 1	1.584	1.090–2.302	***0.016***	1.526	1.045–2.229	***0.029***
	Group 2	2.625	1.753–3.928	***<0.001***	2.274	1.502–3.444	***<0.001***

Significant findings (p < 0.05) are expressed in bold and italic.

KPS, Karnofsky performance status; IDH-1, Isocitrate dehydrogenase-1; NLR, neutrophil-to-lymphocyte ratio; LMR, lymphocyte-to- monocyte ratio; CONUT, Controlling Nutritional Status; NPS, Naples prognostic score.

### Prognostic model based on NPS and CONUT

Prognostic models were conducted to further compare the prognostic ability between NPS and CONUT score (**Table 3**). The basic model constituted of independent variables from multivariate Cox regression except NPS and CONUT score. The results indicated that the prognostic ability of the basic model with either NPS (C-index, 0.645; AIC, 2,332.11) or CONUT (C-index, 0.624; AIC, 2,343.53) was superior to the basic model alone (C-index, 0.614; AIC, 2,347.70). Among these models, Model NPS had the largest C-index and the lowest AIC, which indicated the strongest prognostic capability and best model-fitting.

**Table 3:** Prognostic models with NPS and CONUT score for GBM patients.

Clinical Variables		Prognostic Models								
		Basic Model			Model CONUT			Model NPS		
		HR	95% CI	*p*-value	HR	95% CI	*p*-value	HR	95% CI	*p*-value
**Age at diagnosis**	<55	Reference			Reference			Reference		
	≥55	1.681	1.290–2.191	***<0.001***	1.732	1.325–2.264	***<0.001***	1.632	1.250–2.131	***<0.001***
**Adjuvant therapy**	Yes	Reference			Reference			Reference		
	No or undone	1.763	1.303–2.387	***<0.001***	1.748	1.291–2.368	***<0.001***	1.702	1.256–2.306	***0.001***
**IDH-1**	Mutant	Reference			Reference			Reference		
	Wild type	1.871	1.216–2.878	***0.004***	1.853	1.204–2.850	***0.005***	1.802	1.171–2.773	***0.007***
**CONUT score**	Normal				Reference		***0.017***			
	Light				1.365	1.002–1.859	***0.049***			
	Moderate				1.981	1.224–3.205	***0.005***			
**NPS**	Group 0							Reference		***<0.001***
	Group 1							1.550	1.064–2.257	***0.022***
	Group 2							2.401	1.595–3.613	***<0.001***
	**C-index**	0.614			0.624			0.645		
	**AIC**	2,347.70			2,343.53			2,332.11		

Significant findings (p < 0.05) are expressed in bold and italic.

IDH-1, Isocitrate dehydrogenase-1; CONUT, Controlling Nutritional Status; NPS, Naples prognostic score; HR, hazard ratio; CI, confidence interval; C-index, Harrell’s concordance index; AIC, Akaike information criterion.

## Discussion

In recent years, NPS has been widely researched in patients with malignancies, such as colorectal cancer, pancreatic cancer, endometrial cancer, lung cancer, gastric cancer, and esophageal squamous cell carcinoma (18–20, 28–30). These studies have found that preoperative NPS served as a reliable indicator to effectively predict prognosis mainly including OS and progression-free survival (PFS). In the current study, we attempt to explore the prognostic significance of preoperative NPS in newly diagnosed GBM patients. The results from our research are consistent with previous research, which indicates that NPS is an independent prognostic predictor.

Inflammation and malnutrition are basic characteristics for patients with malignancies. Tumor microenvironment, which is largely orchestrated by inflammatory cells, is an indispensable participant in the neoplastic process, fostering proliferation, survival, and migration. Many cancers arise from sites of infection, chronic irritation, and inflammation (8, 31). Changes in systemic inflammation could reflect the progression of local inflammation in tumors or adjacent to tumors; thus, researchers use peripheral blood immune cells to evaluate the progression of disease. In fact, these methods are also applied in patients with chronic medical disease such as coronary heart disease, systemic lupus erythematosus, and end-stage renal disease (32–34). The representative blood inflammatory markers mainly include leukocyte, neutrophil, lymphocyte, platelet, monocyte, and their combinations like neutrophil-to-lymphocyte ratio, platelet-to-lymphocyte ratio, and lymphocyte-to-monocyte ratio, which have been widely used in estimating conditions of diseases.

Nutrition status plays different roles in tumorigenesis and disease progression. On the one hand, nutrition and dietary factors have been associated with cancer risk. Nutrition/dietary components are likely to have an effect on an individual’s risk of cancer and that the mechanism by which cancer risk is affected is likely to be through epigenetic modification of an individual’s genome (35, 36). On the other hand, endless growth of malignancies has a tendency to deplete nutrients and leads to malnutrition conditions, and malnutrition can impact disease progression and survival in cancer patients (37). Various peripheral blood nutritional parameters have been reported to be associated with prognosis in patients with different malignancies. Among these markers, serum albumin concentrate and prognostic nutritional index (PNI) that contains albumin and lymphocyte have been used frequently (38–40). High concentration of nutritional markers, which indicates better nutritional status, usually relates to favorable prognosis. Cholesterol is an uncommon nutritional marker and is vital for the survival and growth of mammalian cells. It has been reported that cholesterol concentrate is significantly correlated with the incidence and progression of various malignancies like prostate cancer and breast cancer (41–44). As an indicator of nutrition, however, high cholesterol concentrate usually has been associated with favorable prognosis in cancer patients (45–47).

There has been a close relationship between systemic inflammatory response and nutrition (48). Nutritional status affects circulating immune cells, especially T cells, in population, metabolism, and function; hence abnormal nutritional conditions would break the balance of systemic inflammation (49). The combination of systemic inflammatory markers and nutritional markers to predict prognosis and progression of disease has been widely used and proved to be useful, and the representatives include Glasgow prognostic score (GPS), CONUT, and NPS (16, 21, 50). Other different combinations of two kinds of markers are also determined to have effective prognostic ability in tumors (51, 52). Some of these prognostic scores like GPS and CONUT are also researched in patients with GBM, and the results are consistent with those of previous studies (25, 53).

There are still some limitations in our research. First, it is a single-center retrospective clinical research, and multi-center collaborations and prospective experimental design are needed to verify the results. Second, it is hard for us to build a validation cohort due to the relatively small sample size. Third, patients lost to follow-up may cause selection bias in analysis. Fourth, some important glioma-related biomarkers like O6-methylguanine-DNA methyltransferase (MGMT) methylation and telomerase reverse transcriptase (TERT) mutation status were not included in the study due to incomplete pathological information.

## Conclusion

To our knowledge, this is the first study to evaluate the prognostic role of preoperative NPS in newly diagnosed GBM patients. In the current study, we find NPS as an independent prognostic indicator for patients with newly diagnosed GBM patients, and the prognostic ability of NPS is superior to a similar prognostic score, CONUT. This easily acquired preoperative prognostic score has the potential to be used in clinical work, and will be verified in future research.

## Data availability statement

The raw data supporting the conclusions of this article will be made available by the authors, without undue reservation.

## Ethics statement

The studies involving human participants were reviewed and approved by Sichuan University. The patients/participants provided their written informed consent to participate in this study.

## Author contributions

Conception and design: YL, JL, and WY. Provision of study materials or patients: JL, YY, and TL. Collection and assembly of data: MZ, ZW, and WY. Data analysis and interpretation: JL and YY. Manuscript writing: All authors. Final approval of manuscript: All authors.

## Funding

This study was supported by the Key Research and Development Item from the Department of Science and Technology of Sichuan Province, China (No. 2017SZ0006).

## Acknowledgments

We would like to express our gratitude to all the colleagues who have assisted clinical data collection. We would like to thank all the glioma patients who were admitted in our hospital who have contributed to the development of medicine.

## Conflict of interest

The authors declare that the research was conducted in the absence of any commercial or financial relationships that could be construed as a potential conflict of interest.

## Publisher’s note

All claims expressed in this article are solely those of the authors and do not necessarily represent those of their affiliated organizations, or those of the publisher, the editors and the reviewers. Any product that may be evaluated in this article, or claim that may be made by its manufacturer, is not guaranteed or endorsed by the publisher.
